# Landscape, barriers, and facilitators of scientific productivity in schizophrenia research in Southeast Asia: A bibliometric analysis

**DOI:** 10.1016/j.amsu.2022.104330

**Published:** 2022-08-10

**Authors:** Mary Nadine Alessandra R. Uy, Ourlad Alzeus G. Tantengco

**Affiliations:** College of Medicine, University of the Philippines Manila, Ermita, Manila, Philippines

**Keywords:** ASEAN, Citation analysis, Mental health, Psychiatry, Scientometrics

## Abstract

**Background:**

Schizophrenia research has significantly grown in the past years. However, there is no comprehensive evaluation of schizophrenia research publications from Southeast Asia (SEA). Thus, this study determined the characteristics and trends of published articles about schizophrenia in SEA through a bibliometric analysis.

**Methods:**

A database search on schizophrenia research in SEA countries was performed using the Scopus databases from 1973 to 2021. Bibliometric information was obtained from Scopus, and network visualization was conducted using VOSviewer software.

**Results:**

There were 1068 articles on schizophrenia from SEA from 1973 until 2021. Schizophrenia research outputs from SEA started to increase from 2000 onwards. Singapore, Malaysia, and Thailand were the most productive countries in schizophrenia research and had the most collaborations. Most schizophrenia research in SEA was published in Asia- or SEA-based journals. The research hotspots for schizophrenia in SEA included treatment, pathophysiology, symptomatology, and psychological and social aspects of schizophrenia. Lastly, correlation analysis showed that gross domestic product per capita, research and development (R&D) expenditures, number of researchers in R&D, number of physicians, and international research collaborations were significantly correlated with higher research productivity and scientific impact in schizophrenia research.

**Conclusion:**

In conclusion, this study showed the trends and gaps for research in SEA and the socioeconomic factors correlated with research productivity for schizophrenia in SEA. This study emphasized increasing financial support and collaborations for schizophrenia research to improve research productivity in schizophrenia in the SEA region.

## Highlights

• There were 1068 articles on schizophrenia from Southeast Asia from 1973 until 2021.
• Southeast Asian countries only contributed 1.09% of the global research outputs on schizophrenia.
• Singapore, Malaysia, and Thailand were the most productive countries in schizophrenia research.
• Most schizophrenia research in Southeast Asia was published in regional or local journals.
• Research funding, number of researchers and physicians, and collaboration increased research productivity in schizophrenia.

## 1 Introduction

Mental health condition has been increasingly on the rise worldwide. One of these conditions is schizophrenia, a severe psychiatric disorder with the hallmark symptom of psychosis characterized by hallucinations and delusions [1]. It is commonly associated with impairments in social and occupational functioning [1]. According to the World Health Organization, this disease is one of the most disabling medical disorders, with schizophrenia causing 1 in 5 years lived with disability [2]. It also has an enormous economic burden on the country's healthcare system. The estimated annual costs for schizophrenia in the country ranged from US$94 million to US$102 billion, approximately 0.02%–1.65% of the gross domestic product [3].

The prevalence of schizophrenia is estimated to be approximately 1 in 300 people globally [4]. Patients are 2–3 times more likely to die early than the general population [4]. This disease is affected by various exposures such as urbanicity and economic status. The incidence of schizophrenia is higher in urban settings and developed nations [5]. However, this should be taken with caution because underreporting cases could be in developing countries. Furthermore, there is a lack of data from low-versus higher-income nations. There is a substantial variation in the long-term course and outcome and morality across different Asian countries, and the reason for this remains explored [6]. Hence, more study is needed in Southeast Asia, where most countries are still developing nations.

Schizophrenia research has significantly grown in the past years. Previous bibliometric analysis showed an increasing trend in schizophrenia research publications worldwide [[7], [8], [9]]. However, there is no comprehensive evaluation of schizophrenia research publications from SEA countries. Determining the evolution and dynamics of research publications in this field can help determine essential areas and gaps for future research on schizophrenia in SEA. Providing timely and evidence-based information about mental health diseases in SEA is necessary, especially during the COVID-19 pandemic [10]. Thus, we performed a bibliometric analysis to assess the characteristics and trends of published articles on schizophrenia in SEA. This research also identified socioeconomic factors contributing to research productivity and the impact of schizophrenia research in SEA.

## 2 Methods

### 2.1 Study selection

A database search was performed using the Scopus database. We used the search term “schizophreni*” and it was only searched for in the article title field. We limited the search to Southeast Asian countries, particularly Brunei, Cambodia, Indonesia, Lao PDR, Malaysia, Myanmar, Philippines, Singapore, Thailand, and Vietnam. All electronic searches were performed on December 12, 2021.

### 2.2 Data collection

A total of 1068 articles were obtained from our search in the Scopus database. The following information was obtained for each article: authors, year of publication, title, journal, institution, country, title, keywords, and citation frequency. These were used to investigate the knowledge domain and development trends of schizophrenia research worldwide.

### 2.3 Statistical analysis

The correlation analysis was conducted in GraphPad Prism version 7 (GraphPad Software, San Diego, CA). The visualization of collaboration networks of countries and keywords related to schizophrenia was conducted using VOSviewer version 1.6.16 (Leiden University, Leiden, Netherlands) [11].

## 3 Results

### 3.1 The annual number of schizophrenia publications

**Fig. 1:**
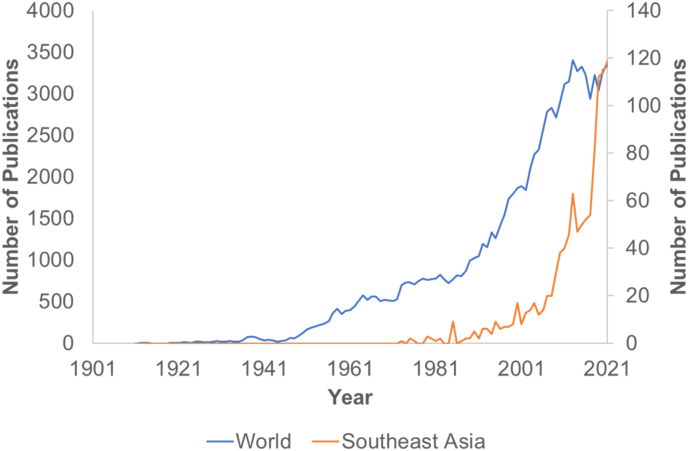
Annual number of published articles relating to schizophrenia in Southeast Asia from 1979 to 2021.

There were 1068 articles on schizophrenia from SEA from 1973 until 2021. Schizophrenia research outputs from SEA increased from 2000 onwards (Fig. 1). Despite the significant increase in schizophrenia research, SEA countries still lagged behind others. SEA countries only contributed 1.09% of the global research outputs on schizophrenia. Moreover, SEA countries started publishing schizophrenia research in 1973, whereas other countries began as early as 1911.

### 3.2 Schizophrenia publications by country

**Fig. 2:**
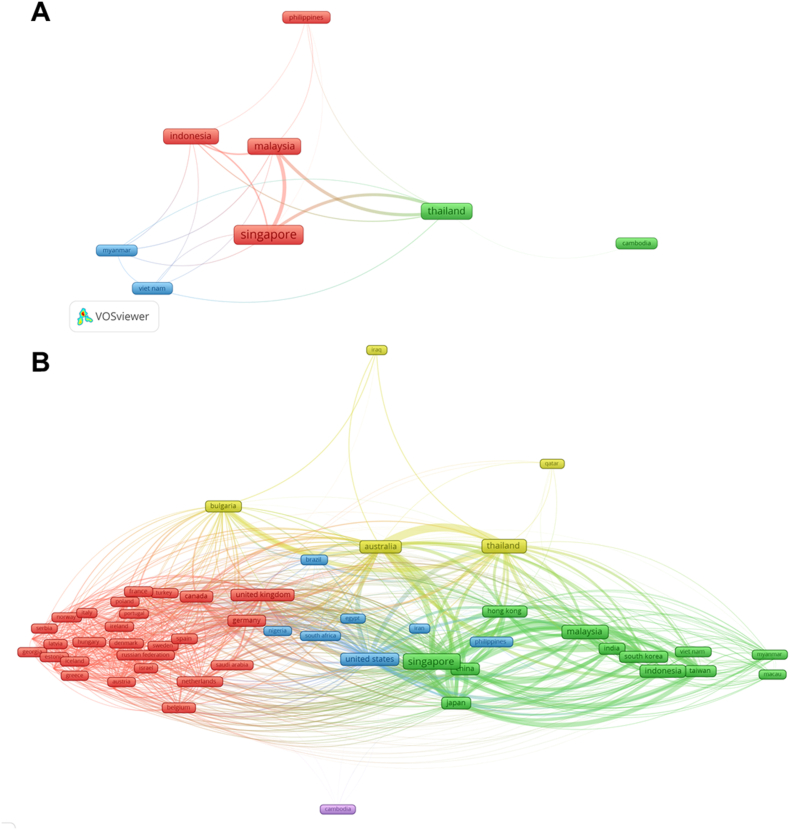
Network visualization of international collaboration for schizophrenia research within Southeast Asia (A) and all over the world (B). The size of the frame is proportional to the number of links/collaborations of each country in the cooperation network. The thickness of the lines indicates the strength of the connection between countries/regions.

The total publications, total citation (TC), citation per publication (CPP), and h-index of SEA countries for schizophrenia research outputs were presented in Table 1. Singapore published the most schizophrenia research among the SEA counties and received the highest citations. Malaysia, Thailand, and Indonesia also contributed significantly to schizophrenia research outputs in SEA. Brunei Darussalam, Laos, and Timor Leste did not have research outputs on schizophrenia. We observed that SEA countries collaborated with other countries within SEA for schizophrenia research. They also had a lot of collaborations with authors from different parts of the world (Fig. 2). Singapore collaborated with 55 countries, Malaysia with 43, and Thailand with 37.

**Table 1:** Citation analysis of schizophrenia research published from Southeast Asian countries from 1973 to 2021.

Country	TP	TC	CPP	*h*-index
Singapore	441	14403	32.65986	46
Malaysia	253	2805	11.08696	26
Thailand	241	3556	14.75519	30
Indonesia	187	1081	5.780749	18
Philippines	23	287	12.47826	8
Viet Nam	17	101	5.941176	6
Myanmar	8	73	9.125	6
Cambodia	5	23	4.6	2

TP = Total Papers; TC = Total Citations; CPP = Citations Per Paper.

### 3.3 Schizophrenia publications by the institution

Regarding the number of schizophrenia total publications, the top institutions were from Singapore, Malaysia, and Thailand (Table 2). The Singapore Institute of Mental Health had the highest research outputs (287 research papers), while the National University of Singapore received the highest citations (10442 citations).

**Table 2:** Citation analysis of schizophrenia research published from Southeast Asian universities and hospitals from 1973 to 2021.

Institution	TP	TC	CPP	*h*-index
Singapore Institute of Mental Health	287	9678	33.72125	39
National University of Singapore	212	10442	49.25472	36
Duke-NUS Medical School	71	5850	82.39437	20
Chulalongkorn University	67	1014	15.13433	19
Nanyang Technological University	50	351	7.02	11
NUS Yong Loo Lin School of Medicine	46	1266	27.52174	16
Universitas Sumatera Utara	42	30	0.714286	3
Universiti Malaya	41	428	10.43902	10
School of Medical Sciences, Universiti Sains Malaysia	39	448	11.48718	11

TP = Total Papers; TC = Total Citations; CPP = Citations Per Paper.

### 3.4 Schizophrenia publications by journal

Interestingly, half of the top journals that published schizophrenia research from SEA were Asia- or SEA-based journals (Table 3). The other half were international journals. These journals were the Journal of the Medical Association of Thailand, Asian Journal of Psychiatry, Singapore Medical Journal, Annals of the Academy of Medicine Singapore, and Asia Pacific Psychiatry. Schizophrenia Research published the most significant number of schizophrenia articles from SEA (47 schizophrenia articles), which received 905 citations.

**Table 3:** Top journals that published schizophrenia research from Southeast Asian countries from 1973 to 2021.

Journal	TP	Cite Score	TC	CPP	h-index
Schizophrenia Research	47	6.5	905	19.26	18
Open Access Macedonian Journal of Medical Sciences	40	1.1	16	0.4	2
Psychiatry Research	36	5	565	15.69	12
Journal of the Medical Association of Thailand	30	0.2	215	7.17	9
Asian Journal of Psychiatry	26	4.7	199	7.65	7
Singapore Medical Journal	24	2.5	167	6.96	8
Annals of the Academy of Medicine Singapore	21	1.7	232	11.05	10
International Medical Journal	20	0.4	51	2.55	5
Neuropsychiatric Disease and Treatment	16	3.9	393	24.56	7
Plos One	16	5.3	312	19.5	10
Asia Pacific Psychiatry	15	2.9	89	5.93	7

TP = Total Papers; TC = Total Citations; CPP = Citations Per Paper.

### 3.5 Schizophrenia research trends in SEA

**Fig. 3:**
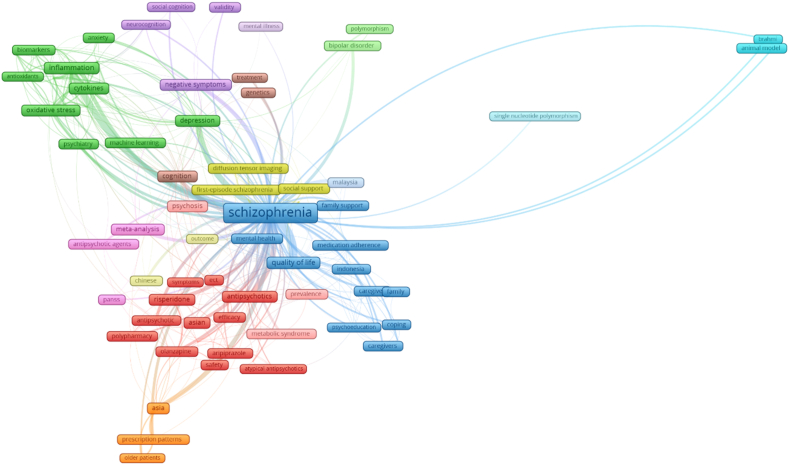
Network visualization of keywords linked to schizophrenia research in Southeast Asia.

Keyword co-occurrence was performed to determine general trends in schizophrenia research in SEA (Fig. 3). The red group represented keywords related to treatments for schizophrenia: antipsychotic, atypical antipsychotic, aripiprazole, ECT, olanzapine, polypharmacy, and risperidone. The green group included keywords associated with the pathophysiology and symptomatology of schizophrenia: anxiety, cytokines, depression, inflammation, and oxidative stress. Lastly, the orange group represented keywords related to psychological and social aspects of schizophrenia: coping, caregiver, family, family support, medication adherence, psychoeducation, and quality of life.

### 3.6 Country-specific socioeconomic factors and correlation with bibliometric indices

Correlation analysis was done to determine the country-specific characteristics (Supplementary Table 1) that were correlated with schizophrenia research productivity in SEA (Table 4). Research & development (R&D) expenditure (*p* = 0.004), researchers in R&D (*p* = 0.005), physicians per 1000 population (*p* = 0.013), and the number of international research collaborations (*p* = 0.0004) were significantly correlated with the total number of publications in schizophrenia in SEA countries. Moreover, GDP per capita (total citations: *p* <0.0001; *h*-index: *p* = 0.011), R&D expenditure (total citations: *p* = 0.001; *h*-index: *p* = 0.004), researchers in R&D (total citations: *p* <0.0001; *h*-index: *p* = 0.005), physicians per 1000 population (total citations: *p* = 0.003; *h*-index: *p* = 0.01), and the number of international research collaborations (total citations: *p* = 0.016; *h*-index: *p* = 0.001) were also correlated with the total citations and *h-*index of SEA countries for their schizophrenia research outputs.

**Table 4:** Correlation analysis between country-specific characteristics and bibliometric indices for schizophrenia research in Southeast Asia.

Country-specific characteristics	Bibliometric indices	r	p-value
Gross domestic product (in USD, billions)	Total publications	0.337	0.415
	Total citations	0.031	0.941
	h-index	0.286	0.492
Gross domestic product per capita (in USD)	Total publications	0.823	0.12
	Total citations	**0.987**	**<0.0001**
	h-index	**0.828**	**0.011**
Population (in millions)	Total publications	−0.141	0.739
	Total citations	−0.37	0.366
	h-index	−0.193	0.647
Research & Development Expenditure (% GDP)	Total publications	**0.877**	**0.004**
	Total citations	**0.925**	**0.001**
	h-index	**0.876**	**0.004**
Researchers in R&D (per million people)	Total publications	**0.867**	**0.005**
	Total citations	**0.983**	**<0.0001**
	h-index	**0.87**	**0.005**
Physicians (per 1000 people)	Total publications	**0.82**	**0.013**
	Total citations	**0.888**	**0.003**
	h-index	**0.837**	**0.01**
International research collaborations	Total publications	**0.944**	**0.0004**
	Total citations	**0.804**	**0.016**
	h-index	**0.962**	**0.0001**
SEA research collaborations	Total publications	0.635	0.091
	Total citations	0.395	0.332
	h-index	0.676	0.066

## 4 Discussion

This bibliometric study of schizophrenia in Southeast Asia showed the following findings: 1) studies on schizophrenia in SEA were low and lagged behind the global research output; 2) Singapore, Malaysia, and Thailand were the most productive countries in schizophrenia research and had the most number of collaborations, and the institutions from these respective countries also contributed the most in terms of research outputs; 3) most of the papers were published within Asia or SEA-based journals; 4) prominent research keywords in schizophrenia included topics about treatment, pathophysiology, and symptomatology, and psychological and social aspects of schizophrenia; and 5) GDP per capita, R&D expenditures, number of researchers in R&D, number of physicians, and international research collaborations were significantly correlated with higher research productivity and scientific impact in schizophrenia research.

To our knowledge, this was the first bibliometric analysis done on schizophrenia in SEA. Publication of studies about schizophrenia lagged globally as it only started during the 1970s, while other countries began publishing as early as 1911. This is not surprising since Asian psychiatry only came into focus during the 1950s [12], unlike in the West, where psychiatric illnesses were studied as early as the 19th century [13]. Nevertheless, research output increased during the 2000s and had a steep peak in 2021. This could be attributed to improved mental health awareness, especially during the COVID-19 pandemic [14].

The majority of the schizophrenia research in SEA was from Singapore, Malaysia, and Thailand. These countries have higher research funding and more physicians and researchers. The results of this study agree with previous bibliometric studies in SEA and showed that these socioeconomic indicators promote research productivity in a country [[15], [16], [17], [18]].

These countries also have the most significant number of collaborations in the field. This is expected as it has been previously established that countries that are more open to collaboration tend to produce more scientific articles than less open countries [18,19]. With this, our study suggests a regional imbalance in the generation of biomedical research. Several factors contribute to the research productivity of a country. As shown in our data, significant correlations were seen in countries with higher GDP, research and development expenditures, and the number of researchers in R&D. This is in agreement with the previous finding that Asian countries with high GDP and that spend more on R&D have a greater scientific impact and research productivity [20].

*Schizophrenia Research* published the most significant number of schizophrenia papers in SEA. This journal targets basic researchers and clinicians and is the most prominent specialist journal in the field. Although this is an international publishing journal, most schizophrenia research in SEA is still published in Asian-based journals such as the *Journal of the Medical Association of Thailand*, *Asian Journal of Psychiatry*, *Singapore Medical Journal*, and the *Annals of the Academy of Medicine Singapore*. Whether international or local, all the top journals that published schizophrenia research from SEA are written in English. This indicates a broader readership of the published papers, hence the higher h-index. This coincides with a previous finding that articles in English are more likely to be cited than those published in other languages [21].

General trends in schizophrenia research can be gleaned through the common keyword co-occurrence associated with schizophrenia. Three clusters are dominant in the keyword visualization - those related to treatments, the disease pathophysiology and symptomatology, and those related to psychological and social aspects of schizophrenia. With this, we can surmise that the research landscape in schizophrenia encompasses a lot of bases - from the micro-level where molecular etiology of the disease is examined as in the keywords clustered under pathophysiology, to the macro level where its societal impact is reviewed as in the cluster related to the psychosocial aspects of schizophrenia. These can be the basis for setting the research agenda of the region for schizophrenia.

There are some limitations to this bibliometric analysis. First, our study was limited to Scopus databases and may have missed other journals from other databases. Our study is also limited to articles published in English. This analysis may have missed research articles published in native languages in SEA countries. Despite these limitations, this study still provides a comprehensive picture of research productivity in Southeast Asia regarding schizophrenia. It has identified research trends and gaps in this field of research.

## 5 Conclusion

In summary, this study showed low schizophrenia research productivity in SEA, but its current trend was increasing. Researchers and institutions from Singapore, Thailand, and Malaysia contributed the most and had the most significant impact on schizophrenia research in SEA. Research productivity in schizophrenia research in SEA was correlated with GDP per capita, R&D expenditure, the number of researchers and physicians, and international research collaborations. The government in SEA countries should consider increasing the funding and support to schizophrenia researchers to improve productivity in this field. Researchers should also foster collaboration with other countries within and outside SEA. Lastly, research trends in schizophrenia are wide range, as shown by the keyword co-occurrence results from this study. This study emphasized increasing financial support and collaborations for schizophrenia research to improve research productivity in schizophrenia in the SEA region.

## Data availability statement

The authors confirm that the data supporting the findings of this study are available within the article and its supplementary materials.

## Provenance and peer review

Not commissioned, externally peer reviewed.

## Ethical approval

Not applicable.

## Sources of funding for your research

This study did not receive any funding.

## Consent

Not applicable.

## Author contribution

OAGT and MNARU conceived the review, contributed to analysis and interpretation of available literature, and prepared the manuscript. All authors contributed to the article and approved the submitted version.

## Registration of research studies

1.Name of the registry: N/A
2. Unique Identifying number or registration ID: N/A
3. Hyperlink to your specific registration (must be publicly accessible and will be checked): N/A

## Guarantor

Ourlad Alzeus G. Tantengco.

## Declaration of competing interest

The authors declare that the research was conducted without any commercial or financial relationships that could be construed as a potential competing interest.
