# Supplementary material for: Landscape, barriers, and facilitators of scientific productivity in schizophrenia research in Southeast Asia: A bibliometric analysis
Source: Ann Med Surg (Lond). 2022 Aug 10;81:104330. doi: 10.1016/j.amsu.2022.104330 (PMC9486613; doi:10.1016/j.amsu.2022.104330)
Supplement: Multimedia component 1 [file mmc1.docx]

**Supplementary Table 1**. Socioeconomic indicators of Southeast Asian countries.

| **Country** | **Gross domestic product (in USD, billions)** | **Gross domestic product per capita (in USD)** | **Population (in millions)** | **Research & Development Expenditure (%GDP)** | **Researchers in R&D (per million people)** | **Physicians (per 1000 people)** | **International Collaborations** | **SEA Collaborations** |
| --- | --- | --- | --- | --- | --- | --- | --- | --- |
| Cambodia | 26.316 | 1,571.92 | 16.741 | 0.05 | 18 | 0.2 | 5 | 1 |
| Indonesia | 1,088.77 | 4,038.40 | 269.603 | 0.08 | 90 | 0.4 | 35 | 6 |
| Malaysia | 336.33 | 10,192.46 | 32.998 | 1.26 | 2052 | 1.5 | 37 | 6 |
| Myanmar | 70.89 | 1,332.55 | 53.199 | 0.16 | 17 | 0.7 | 19 | 5 |
| Philippines | 367.362 | 3,372.53 | 108.928 | 0.14 | 189 | 0.6 | 20 | 4 |
| Singapore | 337.451 | 58,483.96 | 5.77 | 2.19 | 6658 | 2.3 | 55 | 6 |
| Thailand | 509.2 | 7,295.13 | 69.8 | 0.48 | 974 | 0.8 | 43 | 7 |
| Vietnam | 340.602 | 3,497.51 | 97.384 | 0.19 | 114 | 0.8 | 22 | 5 |
